# A sensitive method for atmospheric sulfur dioxide determination by reaction cell inductively coupled plasma mass spectrometer

**DOI:** 10.1098/rsos.251097

**Published:** 2025-11-12

**Authors:** Qi-Xuan Sun, Bo Li, Xiao-Ping Kong, Shang-Qing Zhang

**Affiliations:** ^1^ Henan Institute of Metrology, Zhengzhou, Henan, People’s Republic of China; ^2^ Northeastern University, Shenyang, Liaoning, People’s Republic of China

**Keywords:** sulfur dioxide, air pollutants, collision cell technology inductively coupled plasma mass spectrometer, preconcentration

## Abstract

A sensitive method for sulfur dioxide (SO_2_) determination was developed with reaction cell inductively coupled plasma mass spectrometer (ICP-MS) by preconcentrating SO_2_ from gas samples to solutions. O_2_ flow rate was set at 0.7 ml min^−1^ for optimal signal to noise ratio at *m/z* 48 (^32^S^16^O^+^). Favourable conditions to prepare SO_2_ concentrated solutions including sampling flow rate, the volume of concentrated solution, the number of perfluoroalkoxy alkane (PFA) bubble absorption bottles and the composition of concentrated solution were investigated using SO_2_ in nitrogen gas reference materials within the range of 5.00 parts per billion (ppb) to 1.00 parts per million (ppm). A limit of detection (LOD) of 0.11 ppb was achieved, corresponding to 100 l 5.00 ppb SO_2_ with a sampling flow rate of 0.5 l min^−1^. This method also showed favourable selectivity in the assay of atmosphere SO_2_ level, providing its promising further application in monitoring gaseous environmental pollutants.

## 1. Introduction

Sulfur dioxide (SO_2_) is one of the main pollutants in the atmosphere, which mostly is derived from the combustion of sulfur-containing fuels by anthropogenic activities [1]. Many industrial processes involve the use and emission of SO_2_ such as wine brewing, sewage disposal and food preservation [2]. Besides, volcanic eruptions also emit large amounts of SO_2_ and other sulfur-containing compounds. SO_2_ is a colourless and transparent gas with a strong pungent odour and is often considered toxic and hazardous to human health and the environment. For instance, low-concentration exposure to SO_2_ can cause irritation of eyes, nose, throat, lungs and other mucous tissues, resulting in tearing, nose pain, coughing, asthma, etc., due to its corrosivity and acidity [3]. Furthermore, in the presence of catalysts, SO_2_ can be oxidized to SO_3_ and react with other atmospheric compounds to form acidic particles, leading to air pollution such as acid rain, which can be divided into wet sedimentation when the acidic particles and gases fall to the ground with atmospheric precipitation and dry sedimentation without atmospheric precipitation [4]. Acidic particles may be inhaled into the lungs and pose a serious health risk, while wet sedimentation can cause damage to building structures, mechanical equipment, cultural relic artworks, vegetation and crops [5]. To minimize the above-mentioned harm to human health and ecological environment, it is highly demanded to develop sensitive and selective methods for SO_2_ detection to monitor and control the content of SO_2_ in the atmosphere.

Recently, various analytical techniques have been reported for SO_2_ detection in food, biological materials and environmental samples, including colorimetry [3], fluorimetry [6,7], spectrophotometry [8–11], gas chromatography [12], microfluidic devices [13,14], electrochemical sensors [15,16] and photoacoustic spectroscopy detection [17–20]. SO_2_ chemical sensors involving colorimetric and fluorescent analysis based on specific reactions with SO_2_ or its derivatives usually exhibit poor sensitivity and selectivity, attributed to the interferences from sulfur-containing compounds which result in high background signals. Furthermore, some active gas molecules existing in ambient air during SO_2_ detection may tend to react with sensor materials and contribute to a high limit of detection (LOD). Laser-based photoacoustic spectroscopy technology has been successfully applied in trace gas detection in decades, offering real-time measurements with good sensitivity for a large spectrum of gas species. Apart from all the advantages, the sensor sensitivity is subject to the frequency fluctuations of laser power and physical vibrations of the experimental environment. Besides, the overlap of strong absorption bands with other coexisting ambient air gases should be considered.

In the twentieth century, the absorption-based approaches based on molecular spectroscopy [21] and atomic spectroscopy techniques [22] have been widely employed for atmospheric SO_2_ detection. The classic pararosaniline colorimetric method proposed by West & Gaeke [23] has been continuously improved and used as a reference method in some countries, despite all the complicated procedures. Syty [22] reported a direct and specific SO_2_ determination method by atomic absorption spectroscopy with ultraviolet absorption, showing lower sensitivity compared with the West & Gaeke method. An indirect procedure by flame atomic absorption spectrometry mentioned that the absorbance signal intensity of some alkali earth metal ions shows great dependence on the anions present, which established the basis for sulfuric acid determination [24]. Some titration methods involve the reaction of SO_2_ with oxidants which are commonly applied to high-level SO_2_ determination [25]. Similarly, atmosphere SO_2_ can be collected using hydrogen peroxide and detected by ion chromatography, and the reported SO_2_ concentrations were as low as 5 parts per billion (ppb) for 24 h collection [26]. The traditional absorption-based approaches for SO_2_ determination still require improvement in terms of convenience and sensitivity, since the modern automated monitoring techniques report more than one datum within an hour and the concentration of SO_2_ can be less than 5 ppb [18].

Inductively coupled plasma mass spectrometer (ICP-MS) is well known as a competitive elemental analysis technology in the field of environmental pollution, food and drug safety, life science, geological analysis and so on [27–29]. With a wide element detection coverage, ICP-MS can analyse almost all naturally occurring elements and many artificial radioactive elements. In addition, ICP-MS not only has high sensitivity and selectivity to the target elements, but also has the characteristics of wide dynamic detection range, fast analysis speed and simultaneous multi-element analysis ability [30]. Most metal elements that have much lower first ionization energy than argon tend to reach the ideal ionization efficiency, thus contributing to superior analytical performance [31]. However, non-metal elements like phosphorus and sulfur have small mass and high first ionization energy, exhibiting unfavourable sensitivity consequently. One of the effective solutions is to apply a dynamic reaction cell using He, H_2_, O_2_ and NH_3_ as collision gas or reaction gas, which can even enable the quantification of phosphorus and sulfur levels in single cells on time-resolved mode [32].

Herein, we proposed a convenient and time-saving method for sensitive and selective SO_2_ determination consisting of SO_2_ preconcentration from gas samples to liquid samples and ICP-MS measurement by monitoring the signal intensity at *m/z* 48 (^32^S^16^O^+^). O_2_ was chosen as reaction gas, and O_2_ flow rate was optimized to realize optimal signal to noise (S/N) ratio. In nitrogen gas reference materials, 5.00 ppb to 1.00 parts per million (ppm) SO_2_ were applied to verify the feasibility of the method under different conditions to preconcentrate SO_2_ from gases to solutions within 4 h. This method showed favourable selectivity in the assay of atmospheric SO_2_ level, providing its potential application in monitoring gaseous environmental pollutants.

## 2. Experimental

### 2.1. Materials and chemicals

We purchased 1.00 ppm SO_2_ in nitrogen gas reference material, 10.0 ppm SO_2_ in nitrogen gas reference material, 1.00 ppm hydrogen sulfide (H_2_S) in nitrogen gas reference material and 1.00 μmol mol^−1^ carbonyl sulfide (COS) in nitrogen gas reference material, 100 ppm CO in nitrogen gas reference material, 100 ppm CO_2_ in nitrogen gas reference material, 100 ppm NO in nitrogen gas reference material and 100 ppm NO_2_ in nitrogen gas reference material from Sichuan Dingsheng Technology Co., Ltd. High purity N_2_ (99.999%) was obtained from Henan Yuanzheng Technology Development Co., Ltd. S in water reference material (1000 μg ml^−1^) was purchased from National Standard Material Resource Sharing Platform of China. Nitric acid (65%) was obtained from Sigma-Aldrich.

### 2.2. Instrumentation

ICP-MS analyses were conducted on a single quadrupole-based iCAP RQ inductively coupled plasma mass spectrometer (ICP-MS, ThermoFisher Scientific, Germany) by using O_2_ as reaction gas and monitoring the signal intensity of ^32^S^16^O^+^. A gas dilution system (Henan Institute of Metrology, Henan) was used to dilute gas reference materials to the target concentrations.

### 2.3. Experimental process

#### 2.3.1. SO_2_ enrichment system and purging preparation

Perfluoroalkoxy alkane (PFA) tubes were used to connect a gas dilution system, a SO_2_ in nitrogen gas reference material cylinder, a high purity nitrogen cylinder, a stainless steel needle valve, a PFA bubble absorption bottle and an antiseptic wet gas flow meter. Before sampling, the PFA bubble absorption bottle is not equipped with deionized water or NaOH solution. N_2_ (99.999%) was chosen to purge the gas flow path for 5 min to remove residual gas with a flow rate of 0.5 l min^−1^.

#### 2.3.2. Preparation of SO_2_ concentrated solution samples

Concentrated solution (10–70 ml; deionized water or 5 μmol l^−1^ NaOH solution) was added into the PFA bubble absorption bottle, which was afterwards set on an ice-water bath. After pre-cooling for 10 min, the gas flow rate was adjusted from 0.3 to 0.9 l min^−1^ and the sampling continued for 7–200 min. Finally, SO_2_ concentrated solution in the PFA bubble absorption bottle was collected and stored in a 4℃ refrigerator and detected using ICP-MS within 8 h. SO_2_, 1.00 and 10.0 ppm, in nitrogen gas reference materials were used and diluted with N_2_ (99.999%) by a gas dilution system from 5.00 ppb to 1.00 ppm. All sample solutions were prepared using high-purity deionized water by a Milli-Q reagent water system (Millipore, Bedford, MA, USA). All SO_2_ concentrated solution samples were prepared at atmospheric temperature and pressure.

#### 2.3.3. Inductively coupled plasma mass spectrometry measurements of SO_2_ concentrated solution samples

The introduction of reaction gas O_2_ is conducive to reducing background interference and improving the limit of detection for S measurement by ICP-MS. After tuning with iCAP Q/RQ TUNE solution (1.0 μg l^−1^ Ba, Bi, Ce, Co, In, Li and U in 2% HNO_3_ and 0.5% HCl) supplied by Thermo Scientific, the effect of O_2_ flow rate was investigated from 0.2 to 1.0 ml min^−1^ on the signal intensity of ^32^S^16^O^+^ from 100 μg l^−1^ S standard solution and background. We chose 0.7 ml min^−1^ as O_2_ flow rate to realize optimal instrument performance. Calibration curves were prepared with 0.0, 10, 20, 50, 100, 200 and 500 μg l^−1^ S standard solutions (diluted with 2% HNO_3_). The operating parameters of ICP-MS are listed in electronic supplementary material, table S1.

## 3. Results and discussion

### 3.1. Optimization of O_2_ flow rate

**Figure 1.**
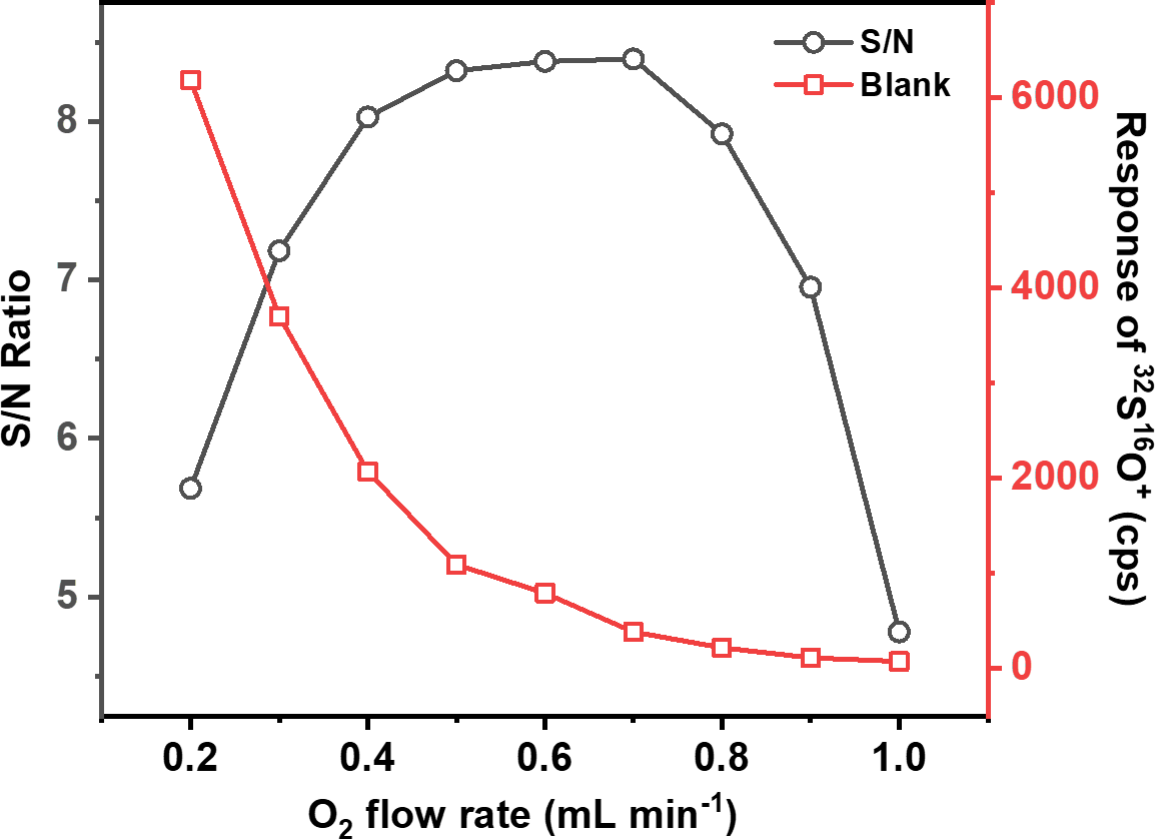
Dependence of ^32^S^16^O^+^ signal intensity in blank solution and S/N ratio on O_2_ flow rate.

Flow rate of reaction gas O_2_ is crucial to the operating parameters of dynamic reaction cell, which in this study was investigated to obtain the ideal S/N ratio. Figure 1 shows the effects of O_2_ flow rate on signal intensity of background in blank solution and on the value of S/N at *m/z* 48 (^32^S^16^O^+^). Obviously, the signal intensity of background at *m/z* 48 (^32^S^16^O^+^) decreased by approximately 85 times with the increase of O_2_ flow rate ranging from 0.2 to 1.0 ml min^−1^. While the value of S/N at *m/z* 48 (^32^S^16^O^+^) increased rapidly with the increase of O_2_ flow rate from 0.2 to 0.5 ml min^−1^, followed by an unremarkable increase with the increase of O_2_ flow rate from 0.5 to 0.7 ml min^−1^, and ended with a rapid decrease with the increase of O_2_ flow rate from 0.7 to 1.0 ml min^−1^. This indicated that an overly high O_2_ flow rate may lead to severe loss of the target ion at *m/z* 48 (^32^S^16^O^+^). Taking all into account, O_2_ flow rate at 0.7 ml min^−1^ was selected to conduct throughout the whole experiment.

### 3.2. Quantification of SO_2_ in gas samples with inductively coupled plasma mass spectrometry measurements

**Figure 2.**
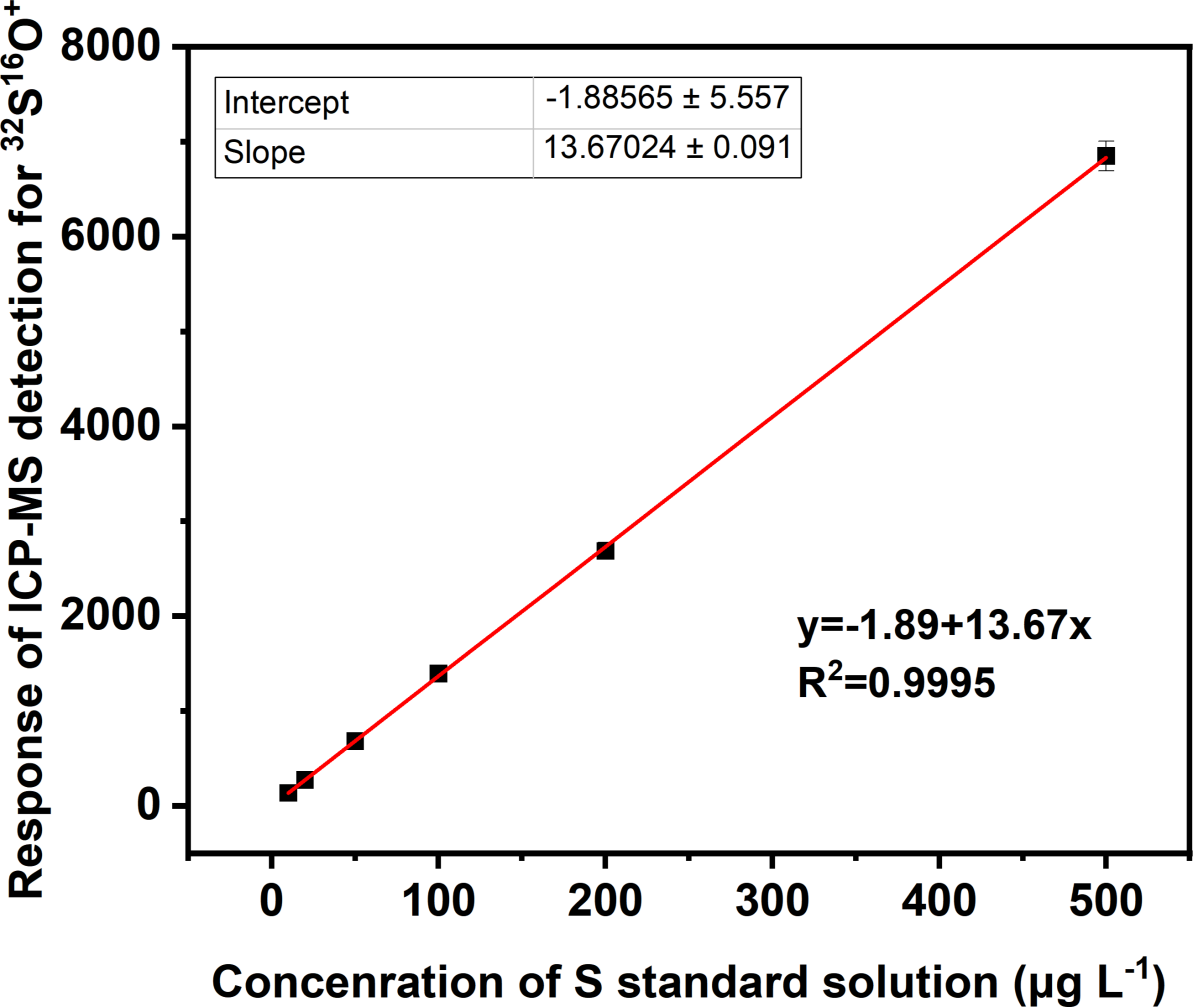
The linear relationship between the response of ICP-MS detection (counts per second, cps) for the ion of ^32^S^16^O^+^ and the concentration of S standard solutions within the range of 10–500 μg l^−1^.

For the quantification of sulfur in the concentrated solution sample, a calibration curve was obtained from a series of S standard solutions within the range of 10–500 μg l^−1^. As shown in figure 2, the detection of ^32^S^16^O^+^ at *m/z* 48 with ICP-MS derived a linear regression equation of counts per second (cps) = −1.89 + 13.67*c*_s_ (*R*^2^ = 0.9995) between the value of cps and sulfur concentration, along with a LOD of 1.5 μg l^−1^. After the quantification of sulfur in the concentrated solution sample, the concentration of SO_2_ in the gas sample was calculated using equation (3.1) deriving from the equation of state of ideal gas,


(3.1) $$V_{0}=\frac{V_{1}\times p_{1}\times 273.15}{1.01\times 10^{5}\times \left(273.15+t_{1}\right)},$$

with *V*_0_ as the volume of SO_2_ in gas sample under standard conditions, l; *V*_1_ as the volume of SO_2_ in gas sample under experimental conditions, l; *p*_1_ as atmospheric pressure under experimental conditions, Pa; *t*_1_ as the ambient temperature under experimental conditions,℃, and equation (3.2),


(3.2) $$x=\frac{c_{s}\times V_{s}\times V_{m}}{V_{0}\times M_{S}}\times 10^{-6},$$

with *x* as the calculated volume fraction of SO_2_ in gas sample; *c*_s_ as the mass concentration of sulfur in the concentrated solution sample, mg l^−1^; *V*_s_ as the volume of concentrated solution, ml; *V*_m_ as molar volume of gas, 22.4 l mol^−1^; *M*_S_ as the relative atomic mass of sulfur.

### 3.3. Influence of operation conditions on SO_2_ absorption efficiency

Conditions to prepare SO_2_ concentrated solution samples such as sampling flow rate, the volume of concentrated solution, the number of PFA bubble absorption bottles, the composition of concentrated solution and the volume fraction of SO_2_ in nitrogen gas reference materials were thoroughly evaluated by the value of assumed absorption efficiency, defined as *η*, which is calculated based on equation (3.3),


(3.3) $$\eta =\frac{x}{x_{R}},$$

where *x* is the volume fraction of SO_2_ in gas sample determined by our proposed method in this work; *x*_R_ is the volume fraction of SO_2_ in nitrogen gas reference materials.

#### 3.3.1. Sampling flow rate

**Figure 3.**
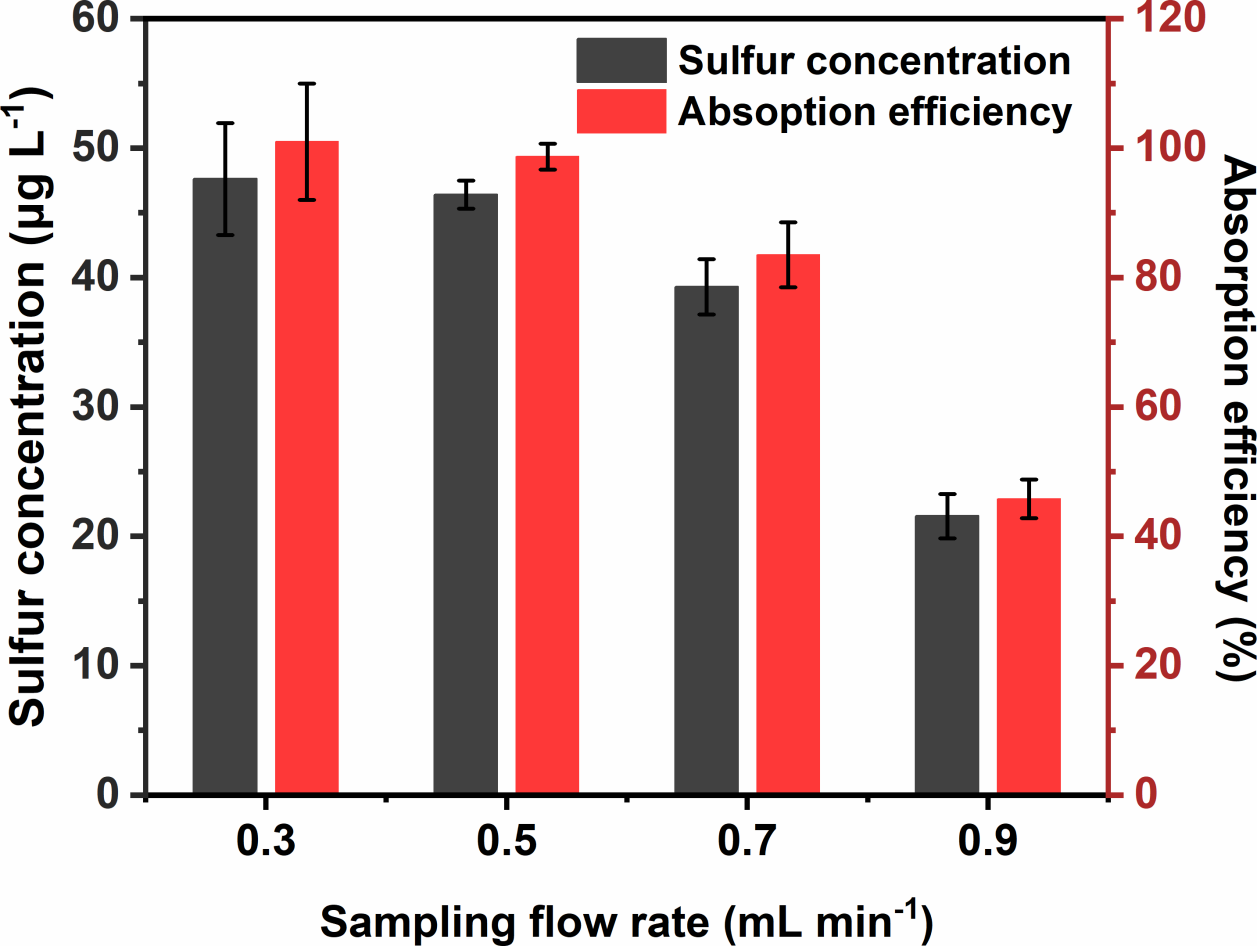
Dependence of sampling flow rate on the mass concentration of sulfur in the concentrated solution sample and absorption efficiency. Concentrated solution: 70 ml deionized water; SO_2_ source: 5 l 1.00 ppm SO_2_; sampling flow rate: 0.3–0.9 l min^−1^.

To investigate the effect of sampling flow rate on SO_2_ concentrated solution samples, 70 ml deionized water was applied to preconcentrate 5 l 1.00 ppm SO_2_ with a flow rate of 0.3, 0.5, 0.7 and 0.9 l min^−1^ in sequence. As shown in figure 3, it was evident that the mass concentrations of sulfur in SO_2_ concentrated solutions and absorption efficiency increased rapidly with the decrease of sampling flow rate from 0.9 to 0.7 l min^−1^, and the increase slowed from 0.7 to 0.3 l min^−1^. The absorption efficiency at 0.9 l min^−1^ was only 45.8 ± 3.6%, which indicated that the sampling flow rate of 0.9 l min^−1^ was too high to realize adequate absorption of SO_2_. In the meantime, the slower the sampling flow rate was, the more time-consuming the preconcentration was, which tended to result in sample contamination regarding massive testing tasks. Therefore, a sampling flow rate of 0.5 l min^−1^ was applied in the ensuing experiment.

#### 3.3.2. The number of perfluoroalkoxy alkane bubble absorption bottles

Fifty millilitres deionized water was applied to preconcentrate 5 l 1.00 ppm SO_2_ with a flow rate of 0.5 l min^−1^. The mass concentration of sulfur in SO_2_ concentrated solutions was obtained from ICP-MS measurement as 122.9 ± 6.7 μg l^−1^, and the responding absorption efficiency was calculated as 94.1 ± 5.1%. Attempting to raise absorption efficiency, an extra PFA bubble absorption bottle filled with 50 ml deionized water was applied as a back bottle next to the PFA bubble absorption bottle in this constructed SO_2_ enrichment system. Disappointingly, the absorption efficiency derived from this two-bottle strategy was 95.2 ± 1.8%, since the amount of SO_2_ in the back bottle was beyond the LOD of sulfur measurement by ICP-MS. Accordingly, one PFA bubble absorption bottle was used in the constructed SO_2_ enrichment system.

#### 3.3.3. The gas dilution system

**Figure 4.**
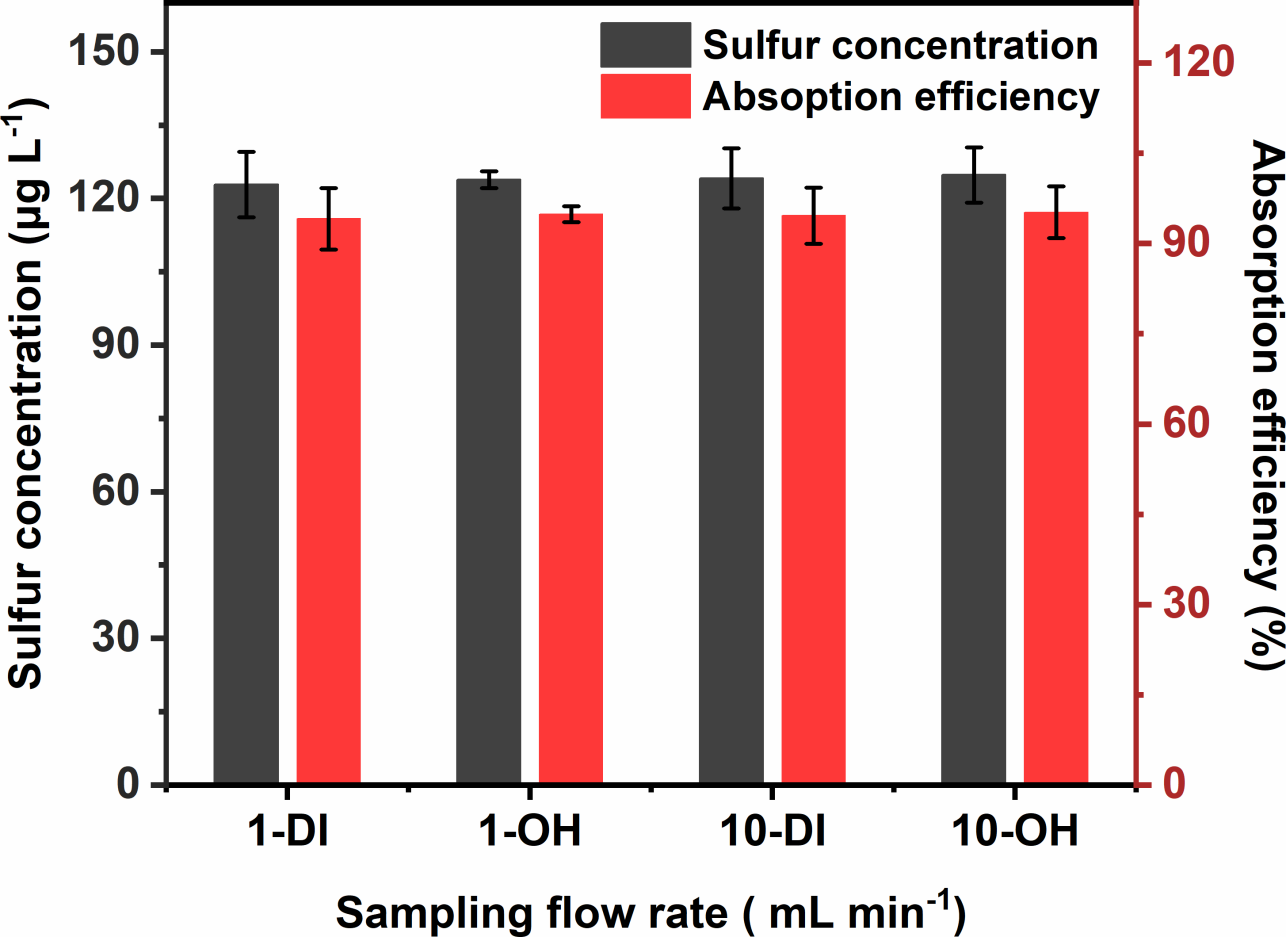
Dependence of the concentrated solution and SO_2_ sources on the mass concentration of sulfur in the concentrated solution sample and absorption efficiency. 1-DI: 50 ml deionized water using 1.00 ppm SO_2_ as direct SO_2_ source; 1-OH: 50 ml 5 μmol l^−1^ NaOH using 1.00 ppm SO_2_ as direct SO_2_ source; 10-DI: 50 ml deionized water using 10.0 ppm SO_2_ as indirect SO_2_ source; 10-OH: 50 ml 5 μmol l^−1^ NaOH using 10.0 ppm SO_2_ as indirect SO_2_ source.

The dilution performance of the gas dilution system was also discussed by comparing the absorption efficiency towards 1.00 ppm SO_2_ in nitrogen gas reference material as direct SO_2_ source and 10.0 ppm SO_2_ in nitrogen gas reference material diluted to 1.00 ppm SO_2_ by gas dilution system with N_2_. Fifty millilitres deionized water was applied to preconcentrate 5 l of the above two SO_2_ sources with a flow rate of 0.5 l min^−1^ and the results are depicted in figure 4. *F*-test and *t*‐test were carried out by assessing whether two results by direct absorption or gas dilution system were significantly different in this case, and the *F* value of 1.19 and *T* value of 1.850 were obtained, indicating no significant difference between the two procedures (*p* = 0.95, *F*_2,2_ = 19.00, *T*_2,2_ = 4.303). In conclusion, the dilution performance of the gas dilution system in our SO_2_ enrichment system was reliable.

#### 3.3.4. The composition of concentrated solution

As a contrast to deionized water, 50 ml 5 μmol l^−1^ NaOH solution was applied to preconcentrate 5 l 1.00 ppm SO_2_ in nitrogen gas reference material as direct SO_2_ source and 10.0 ppm SO_2_ in nitrogen gas reference material diluted to 1.00 ppm SO_2_ by gas dilution system with N_2_ with a flow rate of 0.5 l min^−1^. As shown in figure 4, the mass concentration of sulfur in four concentrated solution samples was determined by ICP-MS as detailed in §2, ranging from 122.9 to 124.8 μg l^−1^ and the values of absorption efficiency were all approximately 95%, which proved the effectiveness of the gas dilution system as well. As a result, desirable absorption efficiency can be achieved by using either deionized water or a 5 μmol l^−1^ NaOH solution in the proposed SO_2_ enrichment system.

#### 3.3.5. The volume of concentrated solution

**Figure 5.**
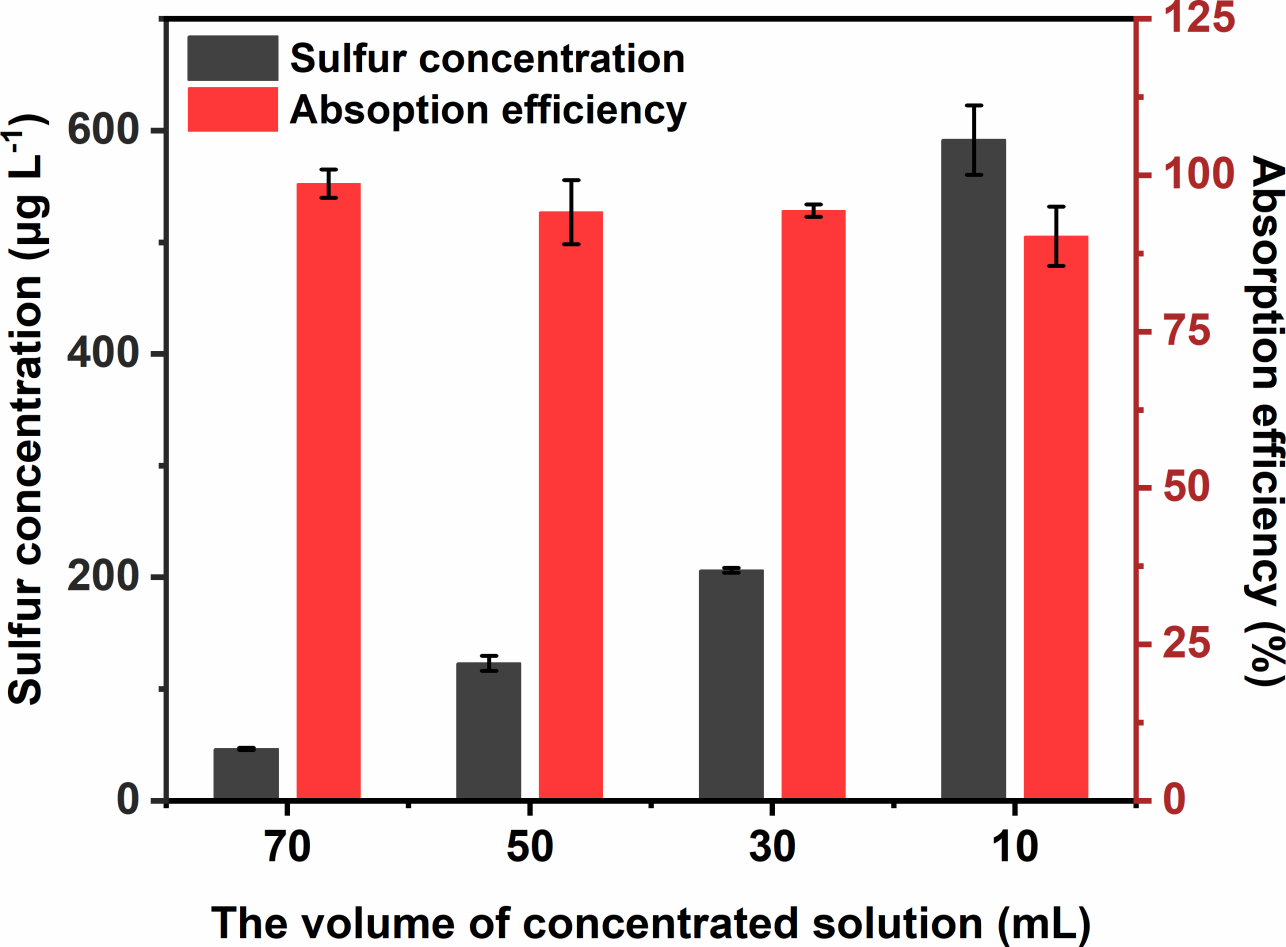
Dependence of the volume of concentrated solution on the mass concentration of sulfur in the concentrated solution sample and absorption efficiency. Concentrated solution: 10–70 ml 5 μmol l^−1^ NaOH; SO_2_ source: 5 l 1.00 ppm SO_2_; sampling flow rate: 0.5 l min^−1^.

To study the influence that the volume of concentrated solution had on absorption efficiency, 70, 50, 30 and 10 ml 5 μmol l^−1^ NaOH solution were applied to preconcentrate 5 l 1.00 ppm SO_2_ in nitrogen gas reference material with a flow rate of 0.5 l min^−1^. As depicted in figure 5, the mass concentration of sulfur in concentrated solution samples increased from 46.4 to 591.6 μg l^−1^, when varying the volume of concentrated solution from 70 to 10 ml, and the values of absorption efficiency were calculated, ranging from 98.7% to 90.3%. This indicated that when the volume of concentrated solution was reduced to 10 ml, absorption for 5 l 1.00 ppm SO_2_ was not as adequate as larger volumes with a flow rate of 0.5 l min^−1^. Besides, the mass concentration of sulfur in 10 ml concentrated solution samples was above the upper limit of the linear range of sulfur measurement by ICP-MS, which required an extra dilution process and amplified measurements errors. Similarly, the volume of concentrated solution larger than 70 ml was not recommended due to the closer mass concentration of sulfur to the lower limit of the linear range of sulfur measurement by ICP-MS, which introduced larger relative errors.

#### 3.3.6. The volume fraction of SO_2_ in nitrogen gas reference materials

The above discussion provided examples for the quantification of 1.00 ppm SO_2_ with ICP-MS assisted by SO_2_ enrichment system, which preconcentrated SO_2_ from gas samples to SO_2_ concentrated solutions. For expanding the application of SO_2_ detection with ICP-MS proposed in this work in trace gas analysis, lower volume fraction of SO_2_ in gas samples at 100, 10.0 and 5.00 ppb was considered. As shown in electronic supplementary material, table S2, recommended conditions were listed for adequate absorption of 100, 10.0 and 5.00 ppb SO_2_, taking all above-discussed affecting factors into account. It is obvious that the preconcentration for 5.00 ppb SO_2_ required a sample volume of 100 l, which took 200 min with a flow rate of 0.5 l min^−1^, providing a possible way to explore trace analysis for SO_2_ in gas samples. However, more improvement for SO_2_ enrichment strategy should be explored for the quantification of SO_2_ at lower concentrations, considering the consumption of sampling volume and sampling time. It is obvious that ICP-MS detection for sulfur in solution samples is not as sensitive as most metal elements. Here we proposed this SO_2_ enrichment system for the preconcentration of SO_2_ from gas samples to concentrated solution samples, and with the assistance of ICP-MS, the LOD of SO_2_ in gas samples was lowered to 0.11 ppb, which is at least one order of magnitude lower than previously reported methods, as shown in electronic supplementary material, table S3. However, a longer sampling time is required, as 4 h for this method, than above-discussed methods. Comparing with traditional absorption-based approaches [23,26], which take 24 h for sampling and lots of chemicals for subsequent procedures, the proposed method is more time-saving, convenient and sensitive. Besides, this method allowed a larger concentration range of SO_2_, enabling more flexible and potential applications.

### 3.4. Selectivity evaluation

**Figure 6.**
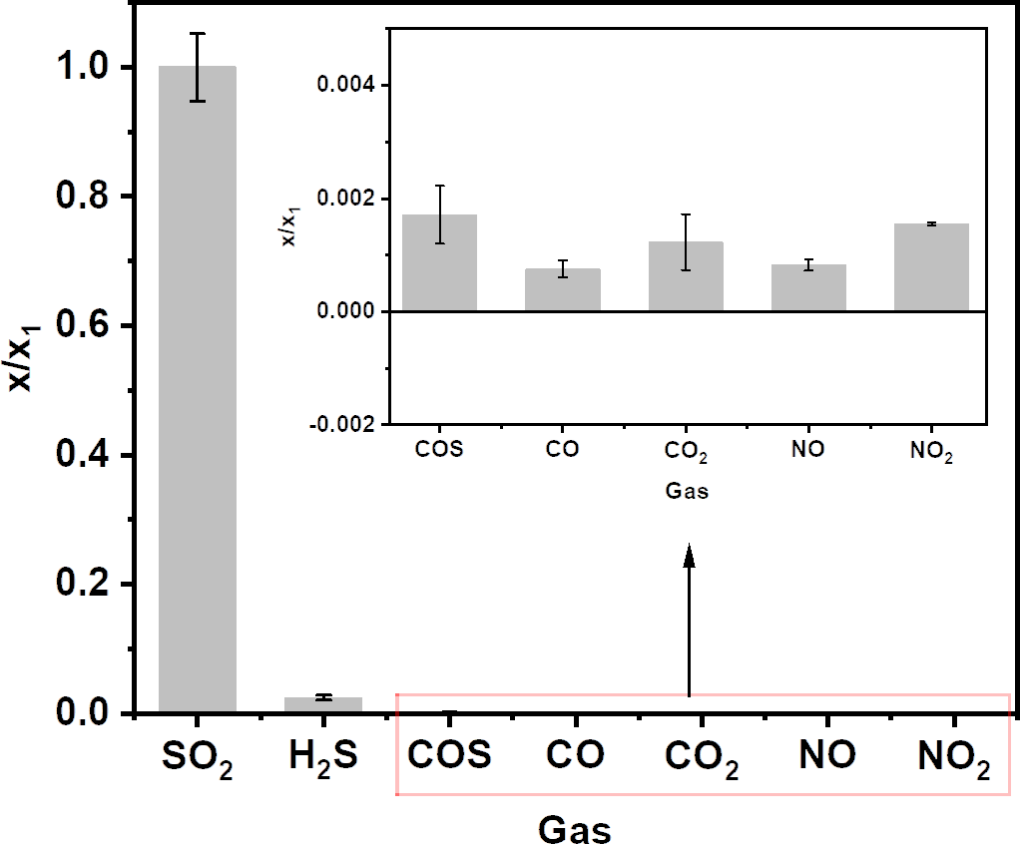
The effects of potential interferences encountered on SO_2_ determination. *x*/*x*_1_ is the ratio of calculated volume fraction of SO_2_ from each gas sample and that from 1 ppm SO_2_ gas reference material. Potential interferences, including 1 ppm H_2_S and COS, as well as 100 ppm CO, CO_2_, NO and NO_2_ gas reference materials, were evaluated. Concentrated solution: 10 ml 5 μmol l^−1^ NaOH; gas volume: 5 L; sampling flow rate: 0.5 l min^−1^.

For the purpose of monitoring SO_2_ level in ambient atmosphere, sulfur-containing gases and other coexisting air pollutants may cause interferences. Thus, similar procedures were conducted to preconcentrate 5 l 1 ppm H_2_S and COS, as well as 100 ppm CO, CO_2_, NO and NO_2_ with 10 ml 5 μmol l^−1^ NaOH solution to evaluate the selectivity of SO_2_ determination with ICP-MS. Figure 6 shows the ratio (*x*/*x*_1_) of the calculated volume fraction of SO_2_ from each gas sample and that from 1 ppm SO_2_ gas reference material. Apparently, the ratio values of H_2_S and COS were smaller than those of SO_2_ at the same condition by over 30 times, probably due to the fact that SO_2_ has good solubility in water. Besides, the interfering effects from CO, CO_2_, NO and NO_2_ at a concentration 100 times higher than SO_2_ on SO_2_ determination were totally negligible, as depicted in the enlarged part in figure 6. Those observations well illustrated favourable selectivity of the proposed method for SO_2_ quantification.

## 4. Conclusions

In this work, a sensitive method for SO_2_ determination with ICP-MS was demonstrated by preconcentrating SO_2_ from gas samples to concentrated solutions, and the LOD of SO_2_ in gas samples was lowered to 0.11 ppb. O_2_ flow rate was optimized to realize optimal S/N ratio at *m/z* 48 (^32^S^16^O^+^), and 5 ppb–1.00 ppm SO_2_ in nitrogen gas reference materials proved the feasibility of the method under different conditions to prepare SO_2_ concentrated solution samples such as sampling flow rate, the volume of concentrated solution, the number of PFA bubble absorption bottles, the composition of concentrated solution and so on. This method also exhibited favourable selectivity in the assay of atmosphere SO_2_ level, providing its potential application in monitoring gaseous environmental pollutants.

## Data Availability

The data supporting this article can be accessed online [33]. Supplementary material is available online [34].

## References

[1] He W, Zhang H, Wang R, Guo J, Zhang Y, Wu K. 2024 Quantitative determination of SO_2_ flux from industrial chimney through machine vision with plume model verification. Measurement **238**, 115255. (10.1016/j.measurement.2024.115255)

[2] Shang Z, Wu M, Meng Q, Jiao Y, Zhang Z, Zhang R. 2024 A near-infrared fluorescent probe for rapid and on-site detection of sulfur dioxide derivative in biological, food and environmental systems. J. Hazard. Mater. **465**, 133165. (10.1016/j.jhazmat.2023.133165) 38061127

[3] Ye Z, Duan C, Sheng R, Xu J, Wang H, Zeng L. 2018 A novel colorimetric and ratiometric fluorescent probe for visualizing SO_2_ derivatives in environment and living cells. Talanta **176**, 389–396. (10.1016/j.talanta.2017.08.054) 28917766

[4] Ebrahimi M, Qaderi F. 2021 Determination of the most effective control methods of SO_2_ pollution in Tehran based on adaptive neuro-fuzzy inference system. Chemosphere **263**, 128002. (10.1016/j.chemosphere.2020.128002) 32846290

[5] Das B, Dursun ÖO, Toraman S. 2022 Prediction of air pollutants for air quality using deep learning methods in a metropolitan city. Urban Clim. **46**, 101291. (10.1016/j.uclim.2022.101291)

[6] Sharma M, Patel C, Samal A, Sriram S, Mukherjee S, Das AK. 2024 Tailoring thiazole decorated polymer with benzoselenadiazole for enhanced SO_2_ sensing. ACS Appl. Polym. Mate. **6**, 6937–6945. (10.1021/acsapm.4c00427)

[7] Zhang T, Zhu L, Ma Y, Lin W. 2020 A near-infrared ratiometric fluorescent probe based on the CN double bond for monitoring SO_2_ and its application in biological imaging. Analyst **145**, 1910–1914. (10.1039/C9AN02322D) 31984996

[8] Yang B, Li W, Xie J, Zhu X, Wang F, Yang Y, Li Z. 2023 Hydrogen sulfide measurement of combustion gaseous product using ultraviolet absorption spectroscopy. Measurement **214**, 112766. (10.1016/j.measurement.2023.112766)

[9] Duan K, Wen D, Ji Y, Xu K, Huang Z, Zhang X, Yao S, Ren W. 2024 Quantum cascade laser absorption sensor for in-situ, real-time and sensitive measurement of high-temperature SO_2_ and SO_3_. Spectrochim. Acta A **309**, 123864. (10.1016/j.saa.2024.123864) 38217990

[10] Li J, Ding Y, Li Z, Peng Z. 2022 Simultaneous measurements of SO_2_ and SO_3_ in the heterogeneous conversions of SO_2_ using QCL absorption spectroscopy. Appl. Phys. B **128**, 61. (10.1007/s00340-022-07776-0)

[11] Yang Y, Li J, Zhang Z, Wang J, Lin G. 2023 Quantitative analysis of SO_2_, NO_2_ and NO mixed gases based on ultraviolet absorption spectrum. Analyst **148**, 6341–6349. (10.1039/D3AN01431B) 37955601

[12] Wang H, Zhou. L, Liu G, Li W, Shi S, Wang Y, Sun J. 2010 Detection of ppb level SO_2_ in H_2_ by an adsorption–desorption gas chromatography method. Int J. Hydrog. Energ. **35**, 2994–2996. (10.1016/j.ijhydene.2009.06.039)

[13] Liu C, Wang Y, Fu L, Yang D. 2017 Rapid integrated microfluidic paper-based system for sulfur dioxide detection. Chem. Eng. J. **316**, 790–796. (10.1016/j.cej.2017.02.023)

[14] Liu Y, Xu X, Chen Y, Zhang Y, Gao X, Xu P, Li X, Fang J, Wen W. 2018 An integrated micro-chip with Ru/Al_2_O_3_/ZnO as sensing material for SO_2_ detection. Sens. Actuat. B Chem. **262**, 26–34. (10.1016/j.snb.2018.01.156)

[15] Hodgson A, Jacquinot P, Hauser P. 1999 Electrochemical sensor for the detection of SO_2_ in the low-ppb range. Anal. Chem. **71**, 2831–2837. (10.1021/ac9812429)

[16] Khan MAH, Rao MV, Li Q. 2019 Recent advances in electrochemical sensors for detecting toxic gases: NO_2_, SO_2_ and H_2_S. Sensors **19**, 905. (10.3390/s19040905) 30795591 PMC6413198

[17] Qi H, Zhao X, Xu Y, Yang L, Liu J, Chen K. 2024 Rapid photoacoustic exhaust gas analyzer for simultaneous measurement of nitrogen dioxide and sulfur dioxide. Anal. Chem. **96**, 5258–5264. (10.1021/acs.analchem.3c05936) 38501986

[18] Yin X *et al*. 2020 ppb-level SO_2_ photoacoustic sensors with a suppressed absorption–desorption effect by using a 7.41 μm external-cavity quantum cascade laser. ACS Sens. **5**, 549–556. (10.1021/acssensors.9b02448) 31939293

[19] Zhao X, Qi H, Wang Z, Ma F, Li C, Guo M, Chen K. 2024 Cantilever enhanced fiber-optic photoacoustic microprobe for diffusion detection of sulfur dioxide. Sens. Actuat. B Chem. **405**, 135340. (10.1016/j.snb.2024.135340)

[20] Zhao X, Zhang Y, Han X, Qi H, Ma F, Chen K. 2024 Pressure-compensated fiber-optic photoacoustic sensors for trace SO_2_ analysis in gas insulation equipment. Anal. Chem. **96**, 10995–11001. (10.1021/acs.analchem.4c01480) 38922420

[21] Scaringelli FP, Saltzman BE, Frey SA. 1967 Spectrophotometric determination of atmospheric sulfur dioxide. Anal. Chem. **39**, 1709–1719. (10.1021/ac50157a031) 6063075

[22] Syty A. 1967 Determination of sulfur dioxide by ultraviolet absorption spectrometry. Anal. Chem. **39**, 1744–1747. (10.1021/ac60331a050)

[23] West PW, Gaeke GC. 1956 Fixation of sulfur dioxide as disulfitomercurate (II) and subsequent colorimetric estimation. Anal. Chem. **28**, 1816. (10.1021/ac60120a005)

[24] Kapitány S, Nagy D, Posta J, Béni Á. 2020 Determination of atmospheric sulphur dioxide and sulphuric acid traces by indirect flame atomic absorption method. Microchem. J. **157**, 104853. (10.1016/j.microc.2020.104853)

[25] Budesinsky BW. 1977 Determination of sulfur dioxide in air. Microchem. J. **22**, 55–59. (10.1016/0026-265X(77)90008-X)

[26] Velásquez H, Ramírez H, Díaz J, González de Nava M, Sosa de Borrego B, Morales J. 1996 Determination of atmospheric sulfur dioxide by ion chromatography in the city of Cabimas, Venezuela. J. Chromatogr. **739**, 295–299. (10.1016/0021-9673(96)00196-3)

[27] Liu ZR, Li XT, Xiao GY, Chen BB, He M, Hu B. 2017 Application of inductively coupled plasma mass spectrometry in the quantitative analysis of biomolecules with exogenous tags: a review. TrAC Trend. Anal. Chem. **93**, 78–101. (10.1016/j.trac.2017.05.008)

[28] Pan H, Feng L, Lu Y, Han Y, Xiong J, Li H. 2022 Calibration strategies for laser ablation ICP-MS in biological studies: a review. TrAC Trend. Anal. Chem. **156**, 116710. (10.1016/j.trac.2022.116710)

[29] Wei X, Lu Y, Zhang X, Chen ML, Wang JH. 2020 Recent advances in single-cell ultra-trace analysis. TrAC Trend. Anal. Chem. **127**, 115886. (10.1016/j.trac.2020.115886)

[30] Clough R, Harrington CF, Hill SJ, Madrid Y, Tyson JF. 2017 Atomic spectrometry update: review of advances in elemental speciation. J. Anal. At. Spectrom. **32**, 1239–1282. (10.1039/C7JA90028G)

[31] Butler OT, Cairns WRL, Cook JM, Davidson CM. 2017 Atomic spectrometry update – a review of advances in environmental analysis. J. Anal. At. Spectrom. **32**, 11–57. (10.1039/C6JA90058E)

[32] Wang H, Wang B, Wang M, Zheng L, Chen H, Chai Z, Zhao Y, Feng W. 2015 Time-resolved ICP-MS analysis of mineral element contents and distribution patterns in single cells. Analyst **140**, 523–531. (10.1039/C4AN01610F) 25407025

[33] Dryad. 2025 Data from: a sensitive method for atmospheric sulfur dioxide determination by reaction cell inductively coupled plasma mass spectrometer. (10.5061/dryad.tb2rbp0dk) PMC12614793 41244058

[34] Sun QX, Li B, Kong XP, Zhang SQ. 2025 Supplementary material from: A sensitive method for atmospheric sulfur dioxide determination by reaction cell inductively coupled plasma mass spectrometer. FigShare. (10.6084/m9.figshare.c.8132391) PMC12614793 41244058

